# Population Subgroups at Risk of Unhealthy Changes in Food and Beverage Consumption During COVID-19 Lockdowns

**DOI:** 10.1007/s12529-023-10165-2

**Published:** 2023-03-06

**Authors:** Leon Booth, Joseph Alvin Santos, Annet C. Hoek, Jacqui Webster, Simone Pettigrew

**Affiliations:** 1 The George Institute for Global Health, University of New South Wales, 1 King St, Newtown, NSW 2042 Australia; 2 UNSW Medicine and Health, University of New South Wales, Sydney, NSW 2052 Australia

**Keywords:** COVID-19, Consumer behaviour, Health behaviour, Alcohol drinking, Diet

## Abstract

**Background:**

Understanding health behaviour changes during the COVID-19 pandemic can assist in developing strategies to promote healthy lifestyles at such times. The aim of this exploratory study was to examine whether the frequency of consuming unhealthy foods and beverages changed during lockdown and whether certain population subgroups were more likely to make such changes.

**Method:**

An online survey was administered to a national sample of 4022 Australian adults (51% female, mean age 48 years). Generalised linear models with generalised estimating equations were used to identify whether demographic characteristics (age, gender, education, presence of children in the household, number of people in the household) and beliefs related to COVID-19 were associated with changes in the frequency of consuming alcohol, sweet snacks, salty snacks, and sugary beverages from pre to during lockdown.

**Results:**

Overall, the frequency of consuming the four assessed unhealthy products did not change during lockdown. However, being male and having children at home were consistently associated with unhealthy changes, whereas believing that alcohol or unhealthy diets would exacerbate COVID-19 severity was linked to a decreased frequency of consuming these products respectively. Age, education, and living with more people were also associated with changes in the frequency of consuming some product categories.

**Conclusion:**

During lockdown, certain population subgroups appeared to be at increased risk of more frequent consumption of unhealthy foods and beverages. Believing certain consumption habits are linked to adverse health impacts of COVID was found to reduce frequency of consumption of related products, presenting a potential focus for future public health actions.

**Supplementary Information:**

The online version contains supplementary material available at 10.1007/s12529-023-10165-2.

## Introduction

The global COVID-19 pandemic has affected many facets of individuals’ lives, including their food and alcohol consumption [1]. Lockdown periods and social distancing measures limit the ways in which alcohol and food products can be accessed and consumed [1], potentially resulting in changes in the patterns of consumption of these products. Greater intake of unhealthy products may impact individuals’ physical and mental health, as well as their COVID-related risks through impaired immune and physiological functioning [2–7]. Greater consumption of unhealthy products during lockdown has been linked to people using alcohol and ‘comfort’ foods as a way to deal with the increased stress experienced [1, 8–10].

In Australia, the context of the present study, lockdowns, and social distancing requirements were periodically enacted during the pandemic as key strategies for reducing the spread of the virus [11]. Research to date suggests this resulted in changes in both food and alcohol consumption patterns among some segments of the Australian population [12]. For example, Australian women were found to be more likely to report increased alcohol consumption during COVID-19 lockdowns [13–15], while young men were found to be more likely to report reduced consumption [16, 17]. These findings have been attributed to females facing additional stressors during lockdown, such as taking on greater caring duties [16].

International studies have linked factors such as such as gender, age, education, living circumstances (being alone or a parent), and COVID-related beliefs to changes in diet and alcohol consumption during lockdowns [1, 8, 10, 18, 19]. While the findings for age, gender, and education have varied across countries [1, 10, 19, 20], it has been proposed the differences may be due to variations in working from home, employment certainty, and childcare responsibilities during lockdowns, which can differ according to one or more of these demographic characteristics [18, 19]. In terms of COVID-related beliefs, believing that a healthy diet will reduce the risk of being infected with COVID-19 has been linked to positive dietary changes during lockdown [20, 21]. This is aligned with the Health Belief Model that emphasises the importance of perceived susceptibility to negative outcomes when making health-related decisions [22].

Research outside of lockdowns suggests that if a person engages in one unhealthy behaviour, they are more likely to engage in multiple unhealthy dietary behaviours, such as the combination of alcohol consumption and eating unhealthy foods [23, 24]. Most work to date on dietary and alcohol consumption changes during COVID-19 has focused on either alcohol or food separately, with little attention given to simultaneous changes across these behaviours and the resulting implications for public health interventions [1]. To address this deficit, the aim of this exploratory study was to examine whether people changed their frequency of consuming unhealthy foods (sweet snacks, salty snacks, sugary beverages, alcoholic beverages) during lockdown and, if so, whether demographic characteristics and COVID-related health beliefs were associated with these changes. Identifying population subgroups most at risk of unhealthy dietary changes can inform the development of interventions designed to promote and facilitate healthy consumption behaviours during lockdowns.

## Methods

### Sample

As part of a larger exploratory project examining dietary changes during COVID-19 lockdowns, 4022 Australian adults were recruited via a web panel provider (Kantar) to complete an online survey. The survey was in field from 16–30 September 2020, which followed a national lockdown between late March and mid May 2020 and other state-based lockdowns of varying durations [16, 25]. During data collection, the state of Victoria still had ‘hard lockdown’ measures in place, with strict limitations on travel distances, time outside of the home, and household visitors [25]. The remaining states and territories were no longer in hard lockdown, but some still had social distancing measures in place [25]. Probability proportional to size sampling was used to recruit a national sample stratified by state, age, and gender (see Table 1). The sample was comparable to the Australian population in terms of gender; however, younger people (18–44 years) were underrepresented while middle-aged people (45–64 years) and those with a tertiary qualification were overrepresented [26]. The study was approved by a university Human Research Ethics Committee.

**Table 1:** Sample profile compared to the Australian adult population

Demographic attribute	Study sample (*n* = 4022)		Australian adult population^a^
	*n*	%	%
*Sex*			
Male	1970	49	49
Female	2052	51	51
*Age*			
Mean (SD)		48 (16)	n/a
18–44	2044	51	44
45–64	1224	30	35
65 +	754	19	21
*Education*			
Non-tertiary	2694	54	72
Tertiary	1328	46	28

Percentages may not add to 100% due to rounding

^a^Age and gender data sourced from Australian Bureau of Statistics [26]. Percentages for education are based on the general Australian population aged 15–74 years using data from the Australian Bureau of Statistics [35]

### Survey Instrument

Respondents reported demographic characteristics including age “*What is your age in years?*” (Open response), gender “*What is your sex?*” (1 = Male, 2 = Female, 3 = Other), education “*What is the highest level of education and training you have completed*” (1 = Never attended school to 9 = Postgraduate university), whether they had children aged < 18 years living in the home “*How many children under the age of 18 live in your household?*” (Open response), and number of people in the household “*How many people, including yourself, live in your household?*” (Open response). COVID-19 health-related beliefs were examined by asking respondents if they agreed that alcohol consumption and an unhealthy diet “*increases the severity of illness from COVID-19*” (1 = Strongly Disagree to 5 = Strongly Agree, or 6 = Don’t know/Unsure).

The pre- and post-lockdown consumption questions were adapted from a previous survey examining dietary changes during COVID-19 lockdowns [27]. Pre-lockdown consumption frequency was assessed by asking “*BEFORE the lockdown, how often did you eat the following (portions of) foods? Please indicate how often you consumed at least one portion of the following foods and drinks*” (1 = Never/Almost Never to 7 = Two or more times per day) for the four dietary outcomes of alcohol, sweet snacks, salty snacks, and sugary beverages. The use of a frequency measure permitted equivalent response options to be applied across the product categories. The next question asked “*DURING the lockdown, how often did you eat the following (portions of) foods? Please indicate how often you consumed at least one portion of the following foods and drinks*” (1 = Never/Almost Never to 7 = Two or more times per day), listing the same product categories as the previous question.

### Data Analyses

Frequency analyses were conducted to identify patterns of change across the four dietary outcomes (alcohol, sweet snacks, salty snacks, and sugary beverages) in terms of the proportion of respondents who increased, decreased, or did not change consumption frequency. To identify variables associated with changes in consumption frequency, generalised linear models with generalised estimating equations were used. The dependent variables were the frequencies of consuming the four product categories, with a separate model run for each product. For each model, the within-subjects factor was time, with pre-lockdown as timepoint 1 and during lockdown as timepoint 2. The independent variables were the demographic characteristics (age, gender, education, children in the household, total number of people in the household) and COVID-19 health-related beliefs (dichotomised into those who agreed the relevant unhealthy dietary behaviour would increase the severity of COVID-19 (response options 4/5) vs. those who selected the remaining response options).

## Results

The frequency analyses demonstrated that most respondents did not change their frequency of consuming alcohol, sweet snacks, and sugary beverages from before to during lockdown (a detailed breakdown is available in Supplementary Table 1). A comparable proportion of respondents increased or decreased their consumption of these products, ranging from 13% each way for alcohol to 21% each way for sweet snacks (see Table 2). Slightly more respondents increased (22%) compared to decreased (18%) their frequency of consuming salty snacks.

**Table 2:** Changes in consumption of unhealthy foods and alcohol during lockdown compared to before lockdown (*n* = 4022)

Change	Alcohol	Sweet snacks	Salty snacks	Sugary beverages
Increase (%)	13^a^	21^a^	22^a^	18^a^
Stay the same (%)	74^b^	58^b^	60^b^	64^b^
Decrease (%)	13^a^	21^a^	18^c^	18^a^

Values with the same superscript letters in each column did not significantly differ at *p* < .05

Consistent with the descriptive analyses, the generalised linear models only revealed a main effect of time for salty snacks, with an increase in frequency of consumption over time (see Table 3). Across the product categories, the models identified several demographic and COVID-19 belief variables associated with increased frequency of consumption. Those with children at home were more likely to increase their frequency of consuming all four product categories (alcohol *B* = .51, sweet snacks *B* = .37, salty snacks *B* = .41, and sugared beverages *B* = .50), whereas those who believed an unhealthy diet would increase COVID-19 severity consumed these products less frequently (alcohol *B* =  − .46, sweet snacks *B* =  − .31, salty snacks *B* =  − .34, and sugared beverages *B* =  − .44). Older people increased their frequency of alcohol consumption (*B* = .01), but reduced their frequency of consuming sweet snacks (*B* =  − .01), salty snacks (*B* =  − .02), and sugared beverages (*B* =  − .02). Male gender was associated with increased frequency of consumption of alcohol (*B* = .65), salty snacks (*B* = .13), and sugared beverages (*B* = .49). A belief that alcohol use would increase COVID-19 severity was associated with decreased frequency of alcohol consumption (*B* =  − .14) but increased frequency for the remaining products (sweet snacks *B* = .16, salty snacks *B* = .20, and sugared beverages *B* = .12). Higher education was linked to more frequent use of alcohol (*B* = .09) and less frequent consumption of sugared beverages (*B* =  − .06). Last, living with more people was linked to more frequent consumption of salty snacks (*B* = .04).

**Table 3:** Generalised estimating equations models’ results for predictors of changes in unhealthy dietary behaviours during lockdown

Variable	Alcohol (*n* = 4022)	Sweet snacks (*n* = 4022)	Salty snacks (*n* = 4022)	Sugared beverages (*n* = 4022)
	*B* [95% CI]	*B* [95% CI]	*B* [95% CI]	*B* [95% CI]
Age	.01 [.01, .01]***	− .01 [− .02, − .01]***	− .02 [− .02, − .02]***	− .02 [− .03, − .02]***
*Sex*				
Male	.65 [.54, .76]***	< .01 [− .09, .09]	.13 [.05, .22]**	.49 [.38, .61]***
Female^a^	-	-	-	-
*Children (*< *18 years) living at home*				
Yes	.51 [.37, .65]***	.37 [.25, .49]***	.41 [.29, .52]***	.50 [.35, .65]***
No^a^	-	-	-	-
Number of people living in household	− .04 [− .09, .01]	.03 [− .01, .08]	.04 [.00, .08]**	< .01 [− .06, .05]
Education (higher education)	.09 [.06, .13]***	− .01 [− .03, .02]	.01 [− .02, .03]	− .06 [− .10, − .03]***
Time	.01 [− .01, .04]	.01 [− .02, .05]	.04 [.01, .07]*	.01 [− .03, .04]
*Agree that factors increase COVID-19 severity*				
Alcohol drinking	− .14 [− .25, − .03]*	.16 [.07, .25]***	.20 [.12, .29]***	.12 [.01, .24]*
Unhealthy diet	− .46 [− .58, − .35]***	− .31 [− .41, − .22]***	− .34 [− .43, − .25]***	− .44 [− .56, − .32]***

*p < .05; **p < .01; ***p < .001

^a^Reference category for nominal variable

## Discussion

The results of the present study are largely consistent with previous Australian findings that overall frequency of consuming alcohol and unhealthy food products did not substantially increase during COVID-19 lockdowns [11, 13, 14, 16, 17, 28]. Like previous research, the findings indicate specific population subgroups exhibited unhealthy changes in dietary patterns [6, 11, 13–16, 29]. These previous studies examined food and alcohol separately, and the present work builds on their findings by identifying males and those with children as being more likely to demonstrate unhealthy changes across several unhealthy product categories during lockdown periods. Consumption patterns according to the remaining demographic variables were less consistent.

In the present study, respondents with children at home were consistently more likely to consume all the examined unhealthy products more frequently, which is aligned with previous findings [28, 30, 31]. Australian parents have self-reported consuming more alcohol to try and manage stress associated with concern for their children and changes to parenting responsibilities during lockdown [30]. Similarly, parents have described snacking more during lockdown due to boredom and increased caregiving-related stress [31]. Some parents in Australia also reported using the extra spare time associated with lockdowns to bond with their children using cooking activities, including preparing and eating unhealthy ‘treats’ such as cakes and biscuits [32].

The finding that males were more likely than females to report increased frequency of consuming alcohol during COVID-19 contradicts previous Australian alcohol research finding the opposite [13–17]. This discrepancy could reflect the different measures used: some of these studies asked respondents whether they consumed more, less, or the same amount of alcohol as they did before lockdown [13–15] and others estimated the quantity of alcohol consumed [16, 17]. Potentially, males are more likely to increase the frequency but not the quantity of alcohol consumed in lockdown compared to females. In the present study, males were also more likely than females to eat more salty snacks and sugary beverages, which conflicts with a study involving a younger student sample that observed the reverse [29].

Given the protracted lockdowns experienced in some parts of Australia and evidence to suggest that unhealthy behaviours developed in response to a traumatic event can last for years [33], this more frequent engagement in multiple unhealthy dietary behaviours by some population segments could increase susceptibility to a range of adverse health conditions. Such conditions include infectious diseases such as COVID-19 through reduced immune function, respiratory impairment, chronic inflammation, greater likelihood of comorbidities, and metabolic and endocrine dysregulation [2–4].

Consistent with international research [21, 34] and the Health Belief Model [22], respondents who recognised the potential for unhealthy foods/beverages or alcohol to increase the risk of severe COVID-19 were less likely to increase their frequency of consuming these products respectively. This suggests a role for greater education to inform the public of how health-related risks can be reduced during lockdowns by managing lifestyle factors, such as by consuming healthy diets and reducing alcohol use. Bhoyroo et al. [12] found that when Australians reflected on their lockdown experiences, they retrospectively desired more public health messaging to encourage healthy behaviours during this time, indicating that such an approach would be considered acceptable. The results of the present study suggest that effective messaging may need to (i) include concrete recommendations and support to minimise any increases in intake of unhealthy foods and beverages, (ii) address certain health beliefs, and (iii) focus on males and those living with children.

### Limitations

This study has several limitations. First, the use of an online panel may have resulted in a skewed sample that differed from the Australian adult population on unassessed characteristics, and the over-representation of middle-aged and tertiary-educated respondents may limit the generalisability of the findings [14]. Second, frequency measures were used to assess consumption, which is less precise than asking respondents to report quantities consumed within specific time periods. Third, respondents were asked to retrospectively recall their frequency of consuming products before and during lockdown, potentially introducing recollection bias. Fourth, the observed relationships cannot be considered causal, limiting the inferences that can be made. Fifth, the use of sex rather than gender is not inclusive of all gender identities. Sixth, there is the potential for respondents to have responded in a way that they perceived to be socially desirable, which could have biased the results. These limitations should be considered in light of the strengths of this study, notably the use of a large and broadly representative sample.

### Conclusion

The present findings indicate that changes in the frequency of consumption of alcohol, sweet snacks, salty snacks, and sugary beverages in the context of a national lockdown were not homogenous among Australian adults. Some population subgroups consumed these products more frequently compared to their pre-COVID-19 consumption patterns, while others consumed them less frequently or remained the same. The increased frequency of consumption of these products was also related to certain health beliefs. This could adversely impact their overall health and reduce their ability to withstand the effects of COVID-19 if infected. Public health responses to pandemics may therefore need to pay particular attention to the potential effects of lockdowns on specific population subgroups that are susceptible to increases in consumption of unhealthy foods and beverages at such times.

### Supplementary Information

Below is the link to the electronic supplementary material.

Supplementary file1 (DOCX 13 KB)

## Data Availability

The data are available on request.
