# Supplementary material for: Population Subgroups at Risk of Unhealthy Changes in Food and Beverage Consumption During COVID-19 Lockdowns
Source: Int J Behav Med. 2023 Mar 6;31(1):163–8. doi: 10.1007/s12529-023-10165-2 (PMC9988201; doi:10.1007/s12529-023-10165-2)
Supplement: Supplementary file 1 — Supplementary file1 (DOCX 13 KB) [file 12529_2023_10165_MOESM1_ESM.docx]

**Supplementary materials**

| Frequency of consumption | Alcohol | | Sweet Snacks | | Salty Snacks | | Sugared beverages | |
| --- | --- | --- | --- | --- | --- | --- | --- | --- |
|  | Before | During | Before | During | Before | During | Before | During |
| Never/Almost never (%) | 26 | 28 | 8 | 11 | 10 | 12 | 28 | 29 |
| Less than once a week (%) | 17 | 15 | 16 | 14 | 20 | 17 | 15 | 14 |
| Once a week (%) | 16 | 15 | 21 | 20 | 23 | 23 | 14 | 14 |
| 2-4 times a week (%) | 19 | 19 | 30 | 29 | 28 | 27 | 16 | 16 |
| 5-6 times a week (%) | 9 | 9 | 11 | 11 | 9 | 10 | 9 | 9 |
| Once a day (%) | 9 | 10 | 11 | 10 | 8 | 9 | 11 | 11 |
| 2 times or more per day (%) | 4 | 4 | 3 | 4 | 2 | 3 | 7 | 8 |

Supplementary Table 1. Frequency of consuming each unhealthy products before and after lockdown.
